# Just Another Storm: Conceptualizing Older Adults’ Perceptions of the COVID-19 Pandemic

**DOI:** 10.1093/geroni/igab046.771

**Published:** 2021-12-17

**Authors:** Christopher Freed, Shoon Lio, Martha Arrieta, Roma Hanks

**Affiliations:** 1 University of South Alabama, Mobile, Alabama, United States; 2 Spring Hill College, Mobile, Alabama, United States

## Abstract

Older adults of color who experience health disparities are especially vulnerable to health and economic adversity related to COVID-19. This study focuses on nine zip codes wherein 70.2% of residents are of African-American descent and an estimated 31.5% of residents live in poverty. To understand the lived experience of the COVID-19 pandemic, perceived challenges of COVID-19, and the dissemination of information related to COVID-19, we collected interview and focus group data in Spring 2020 from fifteen community members, leaders, or advocates. Analyses reveal that older individuals approach the COVID-19 pandemic with familiar disaster mitigation strategies. Other persons perceive the pandemic as another community challenge that African-Americans must confront. Older adults report generational differences in perceptions of the risk of COVID-19 and compliance with health guidelines. Overall, analyses reveal a deeply cultural context for intergenerational responses associated with COVID-19 and a sense of agency among older community leaders as health advocates.

